# Supplementary material for: Unconventional magnetisation texture in graphene/cobalt hybrids
Source: Sci Rep. 2016 Apr 26;6:24783. doi: 10.1038/srep24783 (PMC4844999; doi:10.1038/srep24783)
Supplement: Supplementary Information [file srep24783-s1.pdf]

SUPPLEMENTARY MATERIALS

Unconventional magnetisation texture in graphene / cobalt hybrids

A. D. Vu[1,2], J. Coraux[1,2], G. Chen[3], A. T. N'Diaye[4], A. K. Schmid[3] and N. Rougemaille[1,2,*]
[1]*CNRS, Inst NEEL, F-38000 Grenoble, France*
[2]*Univ. Grenoble Alpes, Inst NEEL, F-38000 Grenoble, France*
[3]*NCEM, Molecular Foundry,*
Lawrence Berkeley National Laboratory,
Berkeley, California 94720, USA
[4]*Advanced Light Source, Lawrence Berkeley National Laboratory,*
Berkeley, California 94720, USA
[*]*nicolas.rougemaille@neel.cnrs.fr*

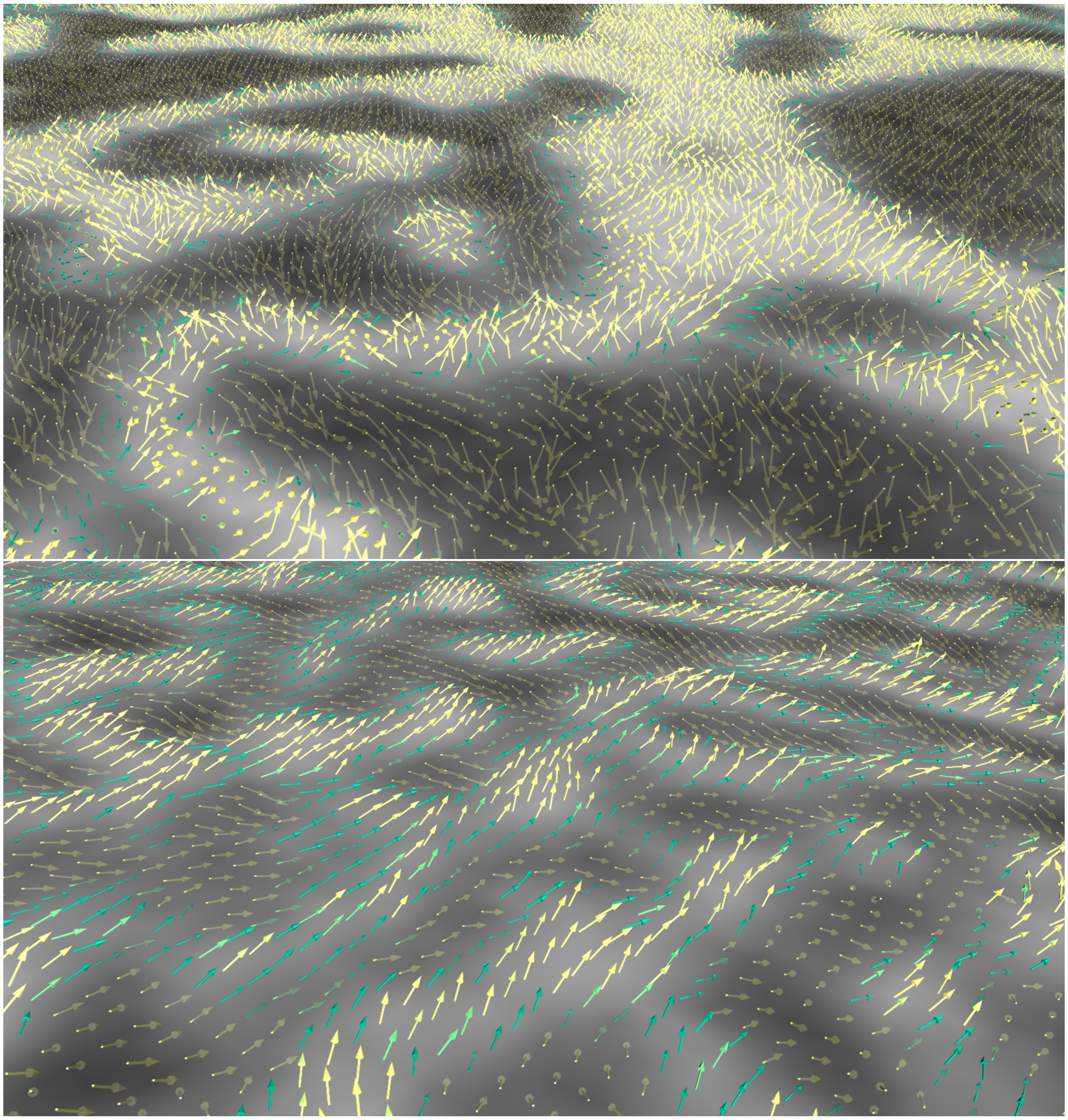

FIG. 1. Three-dimensional, pixel by pixel representation of the magnetisation vector (arrows) in the case of 14 (top) and 18 (bottom) ML of intercalated Co. Black and white contrasts give the out-of-plane component of the magnetisation. Field of view is 3.2×2.4 μm^2.

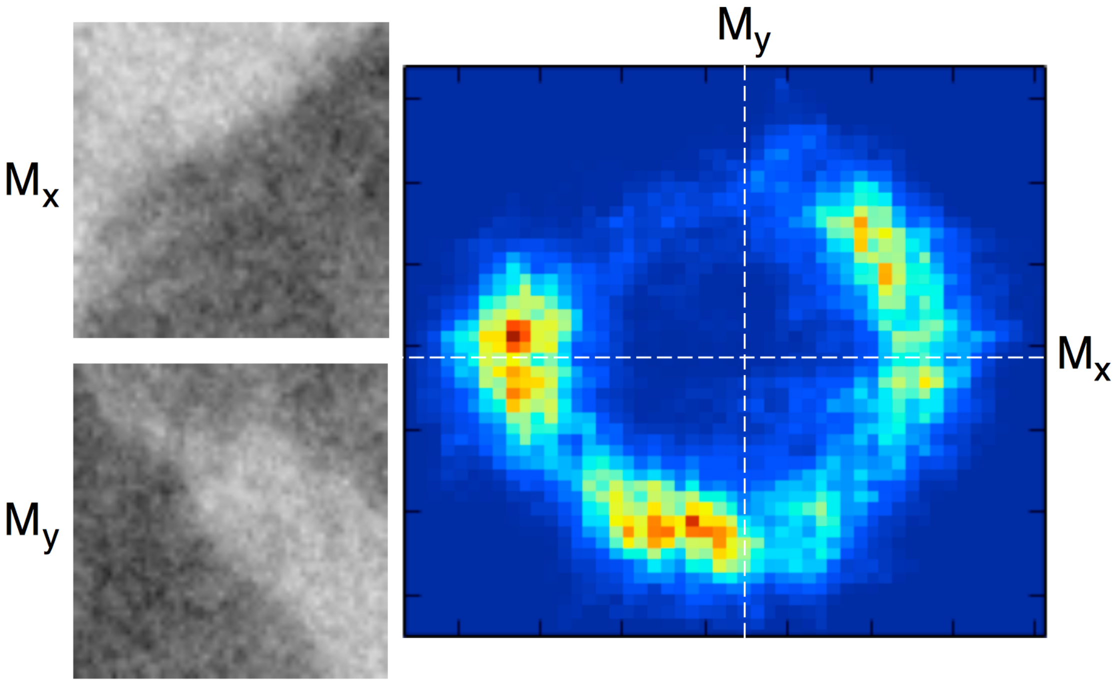

FIG. 2. (left) SPLEEM images for a 20 ML-thick intercalated Co films. (right) Corresponding distribution of the in-plane magnetisation's direction.
